# miR-92b-3p acts as a tumor suppressor by targeting Gabra3 in pancreatic cancer

**DOI:** 10.1186/s12943-017-0723-7

**Published:** 2017-10-27

**Authors:** Manmei Long, Ming Zhan, Sunwang Xu, Ruimeng Yang, Wei Chen, Shilei Zhang, Yongheng Shi, Qiao He, Man Mohan, Qiang Liu, Jian Wang

**Affiliations:** 1 Department of Biliary-Pancreatic Surgery, Renji Hospital, School of Medicine, Shanghai Jiao Tong University, 160 Pujian Road, Shanghai, 200127 China; 2 Department of Pathology, Renji Hospital, School of Medicine, Shanghai Jiao Tong University, 160 Pujian Road, Shanghai, 200127 China; 3 Department of Pathology, Shanghai Ninth People’s Hospital, School of Medicine, Shanghai Jiao Tong University, Shanghai, 200011 China; 4 Department of Biochemistry and Molecular Cell Biology, Shanghai Key Laboratory of Tumor Microenvironment and Inflammation, Institutes of Medical Sciences, Shanghai Jiao Tong University, School of Medicine, Shanghai, 200025 China

**Keywords:** MiR-92b-3p, Pancreatic cancer, Gabra3, Tumor proliferation and metastasis

## Abstract

**Background:**

MicroRNAs (miRNAs) can act as oncogenes or tumor suppressors by controlling cell proliferation, differentiation, metastasis and apoptosis, and miRNA dysregulation is involved in the development of pancreatic cancer (PC). Our previous study demonstrated that Gabra3 plays critical roles in cancer progression. However, whether Gabra3 is regulated by miRNAs in PC remains unknown.

**Methods:**

The expression levels of miR-92b-3p and Gabra3 were measured by quantitative PCR (qPCR), immunoblotting, in situ hybridization (ISH) and immunohistochemistry (IHC). The proliferation rate of PC cells was detected by MTS assay. Wound-healing and transwell assays were used to examine the invasive abilities of PC cells. Dual-luciferase reporter assays were used to determine how miR-92b-3p regulates Gabra3. Xenograft mouse models were used to assess the role of miR-92b-3p in PC tumor formation in vivo.

**Results:**

Here, we provide evidence that miR-92b-3p acted as a tumor suppressor in PC by regulating Gabra3 expression. MiR-92b-3p expression levels were lower in PC tissues than corresponding noncancerous pancreatic (CNP) tissues and were associated with a poor prognosis in PC patients. MiR-92b-3p overexpression suppressed the proliferation and invasion of PC cells in both in vivo and in vitro models. Conversely, miR-92b-3p knockdown induced an aggressive phenotype in PC cells. Mechanistically, miR-92b-3p overexpression suppressed Gabra3 expression, which then led to the inactivation of important oncogenic pathways, including the AKT/mTOR and JNK pathways.

**Conclusion:**

Our results suggest that miR-92b-3p acted as a tumor suppressor by targeting Gabra3*-*associated oncogenic pathways; these results provide novel insight into future treatments for PC patients.

**Electronic supplementary material:**

The online version of this article (10.1186/s12943-017-0723-7) contains supplementary material, which is available to authorized users.

## Background

Pancreatic cancer (PC) is one of the most lethal malignancies in the world, and each year approximately 40,000 patients die from this cancer [1]. In addition to the lack of reliable screening methods for early diagnosis and effective therapies for treatment, highly aggressive metastatic features make the prognosis of PC patients extremely poor [2–4]. Despite our increasing knowledge about the biology of PC, some mechanisms of PC remain unclear, and more effective therapies await discovery. Therefore, identifying novel targets to improve therapeutic strategies for treating PC patients is urgently needed.

Abnormally expressed microRNAs (miRNAs) in various kinds of cancer tissues have been revealed by functional studies; many of these miRNAs have also been identified as tumor suppressors or onco-miRNAs that modulate tumorigenesis, tumor proliferation, apoptosis, metastasis, and chemo-resistance [5–8]. For example, miRNAs, such as miR-19a, miR-30d, and miR-224, can promote tumor invasion, metastasis and tumorigenesis [9–11]. On the other hand, miR-143-3p, miR-141, and miR-505 are potential tumor suppressors [12–14]. miRNAs can influence cancer proliferation and metastasis through multiple pathways, such as the AKT/mTOR and JNK pathways. For instance, miR-1207-5p suppresses AKT/mTOR signaling pathway by targeting fatty acid synthase in hepatocellular carcinoma development [15]. Similarly, miR-21 exerts a protective effect on angiogenesis by regulating MMP2 and MMP9 [16]. Currently, several miRNA-based therapeutics have entered clinical trials; one such example is a mimic of the tumor suppressor miR-34, which has reached phase I clinical trials for the treatment of cancer [17]. Hence, investigations of the complex miRNA-associated pathways may offer insight into identifying new therapeutic targets for treating PC patients.

Gabra3 is a subunit of the gamma-aminobutyric acid (GABA) type A receptor, which is a chloride channel consisting of various subunits [18]. The dysregulation of GABA type A receptors, such as GABA transaminase and GABA transporter, has been suggested to be involved in the brain metastases of breast cancer [19]. It has been proposed that metastatic breast cancer cells might use GABA in stimulating normal neuronal cells to proliferate [19]. In addition, our previous study demonstrated that Gabra3 activates the AKT pathway to promote breast cancer cell migration and invasion [20]. However, whether Gabra3 is also involved in modulating PC development and metastasis remains largely unclear.

Our present study revealed that miR-92b-3p can regulate the proliferation, migration, and invasion of PC cells through modifying Gabra3 expression. By using clinical PC tissue samples, we demonstrated an inverse relationship between miR-92b-3p and Gabra3 expression levels; these levels are also tightly correlated with PC patient prognosis. Collectively, miR-92b-3p directly targeted and reduced Gabra3 expression levels, which led to suppressed proliferation, migration, and invasion in PC cells via the AKT/mTOR and JNK pathways. These findings suggest that the miR-92b-3p/Gabra3 axis is an important regulator of PC development and progression; these results provide potential targets for future PC treatments.

## Results

### MiR-92b-3p expression levels were reduced in human PC tissues and cell lines

**Fig. 1:**
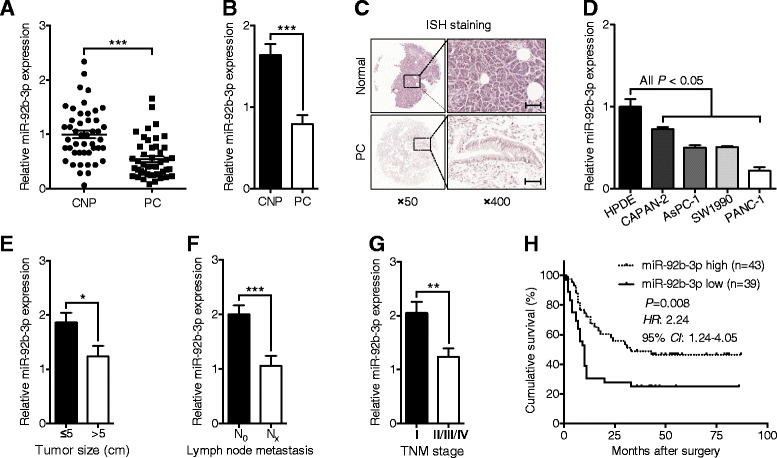
Reduced miR-92b-3p expression levels were found in PC tissues and cell lines. **a** qPCR analyses of the expression levels of miR-92b-3p in 46 fresh PC and CNP tissues. **b** ISH analyses of miR-92b-3p expression levels in 82 FFPE PC and CNP tissues. **c** Representative images of the ISH staining analyses of 82 FFPE PC and CNP tissues using the anti-miR-92b-3p probe. Scale bars: 50 μm. **d** Comparison of miR-92b-3p expression levels in the PC cell lines with those in normal pancreatic epithelial cells by qPCR. **e-g** Association analyses of miR-92b-3p expression levels and tumor size, lymph node metastasis and TNM stage. **h** Kaplan-Meier analyses of postoperative survival in PC patients stratified by miR-92b-3p levels. The *P* value was assessed by log-rank test. *RNU6B* snRNA was used to normalize the qPCR results. Bar, SEM; *P < 0.05; **P < 0.01; ***P < 0.001; Student’s t test

Altered miR-92b-3p expression levels have been found in various types of tumors, but whether miR-92b-3p expression is involved in PC development remains unknown. First, we found that miR-92b-3p expression levels were obviously decreased in 46 fresh PC tissues compared with those in the paired corresponding non-cancerous pancreatic (CNP) tissues (Fig. 1a). In situ hybridization (ISH) staining confirmed remarkably lower miR-92b-3p expression levels in 82 formalin-fixed paraffin-embedded (FFPE) PC tissue samples than the CNP tissues (Fig. 1b-c). Moreover, lower miR-92b-3p expression levels in various PC cell lines than in normal pancreatic epithelial cells was also detected (Fig. 1d). We further studied the relationship between miR-92b-3p expression levels and clinico-pathologic features in 82 PC patients. Reduced miR-92b-3p expression levels correlated well with larger tumor sizes, higher lymph node metastasis rates and advanced tumor/node/metastasis (TNM) stages (Fig. 1e-h). Importantly, cumulative survival rates were significantly lower in PC patients with lower miR-92b-3 expression levels (Fig. 1g). Taken together, these results imply that reduced miR-92b-3p expression levels may play an important role in the development and progression of PC.

### MiR-92b-3p inhibited PC cell proliferation and invasion in vitro

**Fig. 2:**
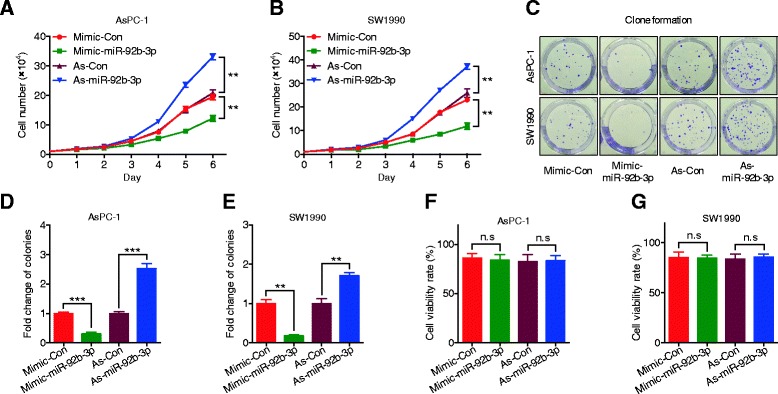
miR-92b-3p inhibited proliferation in PC cells. **a-b** AsPC-1 and SW1990 cell lines were transfected with miR-92b-3p mimic, inhibitor, or negative control, and cell proliferation was measured by MTS assay. **c-e** Colony formation assays in PC cells transfected with miR-92b-3p mimic, inhibitor, or negative control. **f-g** Flow cytometric analyses of Annexin V-FITC staining were used to quantify apoptosis induced by miR-92b-3p. All *n* = 3; bar, SEM; n.s., no significant difference; **P < 0.01; ***P < 0.001; Student’s t test

**Fig. 3:**
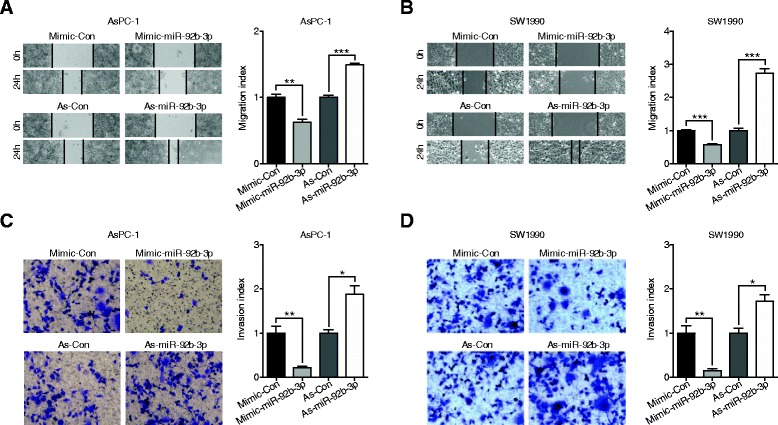
miR-92b-3p suppressed the migration and invasion of PC cells. **a**-**b** Wound-healing assays measured the effect of miR-92b-3p on PC cell motility. **c-d** Transwell assays measured the effect of miR-92b-3p on PC cell invasion. All *n* = 3; bar, SEM; *P < 0.05; **P < 0.01; ***P < 0.001; Student’s t test

To study the potential biological function of miR-92b-3p in PC, MTS assays were conducted to determine the role of miR-92b-3p in modulating PC cell proliferation rates. Compared with the group transfected with the mimic control, AsPC-1 and SW1990 cells transfected with miR-92b-3p mimic had greatly reduced cell numbers according to both MTS assay and colony formation experiments (Fig. 2a-e). Conversely, the opposite effect was shown in PC cells transfected with miR-92b-3p inhibitor (Fig. 2a-e). Cell apoptosis was not affected by altered miR-92b-3p expression levels (Fig. 2f-g). To further examine the influential role of miR-92b-3p in the migration rates of PC cells, wound healing assays were performed. The results showed that AsPC-1 and SW1990 cells with increased miR-92b-3p levels had slower recovery rates than those of control cells; however, decreased miR-92b-3p levels in PC cells led to faster wound closure than in control cells (Fig. 3a-b). Matrigel transwell assays showed a similar phenomenon in which miR-92b-3p overexpression inhibited invasion, and decreased miR-92b-3p levels accelerated AsPC-1 and SW1990 cell invasion (Fig. 3c-d). Together, these results suggest that miR-92b-3p can inhibit the proliferation and invasion of PC cells in vitro.

### MiR-92b-3p bound to the 3′-untranslated region (UTR) of *GABRA3* and inhibited its expression

**Fig. 4:**
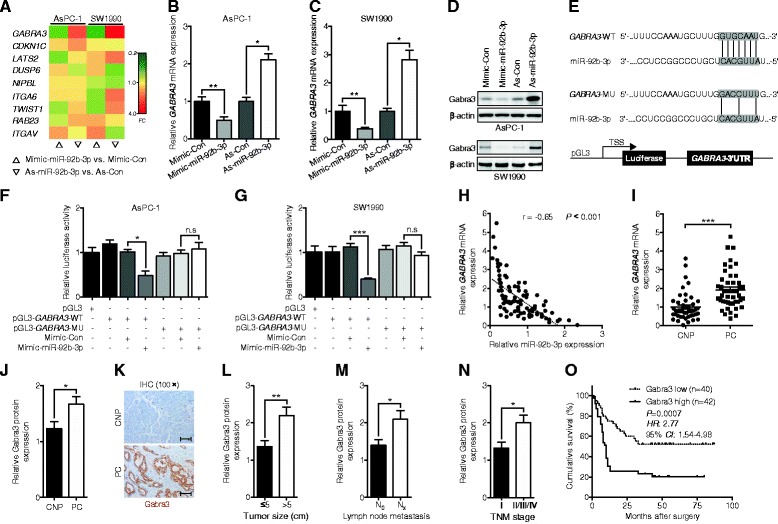
miR-92b-3p directly targeted the 3′-UTR of GABRA3 to suppress its expression. **a** A heat map of the expression changes of 9 candidate genes predicted to be targets of miR-92b-3p in PC cells transfected with miR-92b-3p mimic, antagomir, or negative control. The scale from 0.2 to 4 indicates the intensity of the differential regulation of mRNAs: low expression (green), medium expression (yellow), and high expression (red). FC, fold change. **b-d** qPCR and immunoblotting analyses of the *GABRA3* expression levels in PC cells transfected with miR-92b-3p mimic, antagomir, or negative control. **e** A putative miR-92b-3p-binding site (wild type, WT) existed in the 3′-UTR of Gabra3 mRNA, and a nucleotide mutation (mutant, MU) was created at the binding site. **(F-G)** The relative luciferase activities of either the WT or MU 3′-UTR of the *GABRA3* reporter in combination with the miR-92b-3p mimic in AsPC-1 and SW1990 cells. **h** Pearson χ2 tests were used to analyze the association of miR-92b-3p levels with *GABRA3* levels in 46 pairs of PC and CNP tissues. **i-j** qPCR and IHC analyses of the mRNA and protein levels of *GABRA3* in 46 fresh and 82 FFPE paired PC and CNP tissues. **k** Representative images of IHC staining in the 82 FFPE paired PC and CNP tissues. Scale bars: 100 μm. **l-n** Association of the Gabra3 protein levels with tumor size, lymph node metastasis and TNM stage. **o** Kaplan-Meier analyses of postoperative survival in PC patients stratified by Gabra3 protein levels. The *P* value was assessed by log-rank test. *GAPDH* and *RNU6B* snRNAs were used to normalize the qPCR results. All *n* = 3; bar, SEM; n.s., no significant difference; *P < 0.05; **P < 0.01; ***P < 0.001; Student’s t test

To further elucidate the potential molecular mechanisms involved, a target prediction program (TargetScan Release 7.0: http://www.targetscan.org/vert_71/) [21] was utilized to predict the possible targets of miR-92b-3p. Ultimately, nine candidate genes that could interact with miR-92b-3p were selected for verification. Among them, we found that *GABRA3* was the only one that had similar expression level changes in AsPC-1 and SW1990 cells; *Gabra3* expression levels were increased with antisense-miR-92b-3p transfection and reduced with miR-92b-3p mimic transfection (Fig. 3a-d). Furthermore, Western blot assays confirmed that miR-92b-3p regulated *Gabra3*. To determine whether miR-92b-3p directly regulated *Gabra3* expression, the 3′-UTR of *GABRA3*, which was predicted to interact with miR-92b-3p, was cloned into a pGL3.0 luciferase reporter vector (Fig. 4e). In addition, a reporter carrying a mutated miR-92b-3p binding site was also created (Fig. 4e). Dual luciferase assays showed that while miR-92b-3p suppressed the luciferase activity of the reporter containing the wild type 3′-UTR of *GABRA3* in both AsPC-1 and SW1990 cells*,* the effect was obviously abrogated with the mutated reporter (Fig. 4f-g). Moreover, an inverse relationship between *Gabra3* and miR-92b-3p was also identified in 46 fresh PC and paired CNP tissues (Fig. 4h). Taken together, these results suggest that miR-92b-3p can directly modulate *Gabra3* expression in PC.

To examine the biological significance of Gabra3 in PC, correlations between Gabra3 expression levels and the clinico-pathologic characteristics of PC were analyzed. Via qPCR and IHC assays, we found that both Gabra3 mRNA and protein levels were higer in PC tissues than CNP tissues (Fig. 4i-k). Correspondingly, correlations between reduced Gabra3 expression levels and smaller tumor sizes and lower metastasis rates and TNM stages were also identified in 82 PC patients (Fig. 4l-n). Moreover, poor prognosis was observed in patients with low Gabra3 expression levels (Fig. 4o).

### Gabra3 was involved in the miR-92b-3p-mediated regulation of PC cell growth, migration, and invasion

**Fig. 5:**
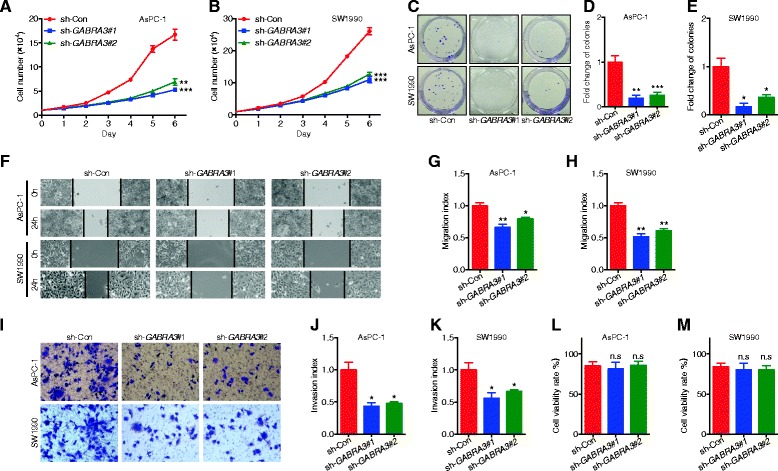
Gabra3 knockdown inhibited cell growth, migration and invasion in PC. **a**-**b** AsPC-1 and SW1990 cell lines were transfected with *GABRA3* shRNAs or a negative control, and cell proliferation was measured by MTS assays. **c-e** Colony formation assays of PC cells transfected with *GABRA3* shRNAs or negative control. **f-h** Wound healing assays were used to assess the motility of cells transfected with Gabra3 shRNAs or negative control. **i-k** Transwell assays were used to assess the invasion ability of PC cells transfected with Gabra3 shRNAs or negative control. **l-m** Flow cytometric analyses of Annexin V-FITC staining were used to quantify apoptosis in PC cells transfected with Gabra3 shRNAs or negative control. All *n* = 3; bar, SEM; Compared with negative control group, n.s., no significant difference, *P < 0.05, **P < 0.01, ***P < 0.001, Student’s t test

**Fig. 6:**
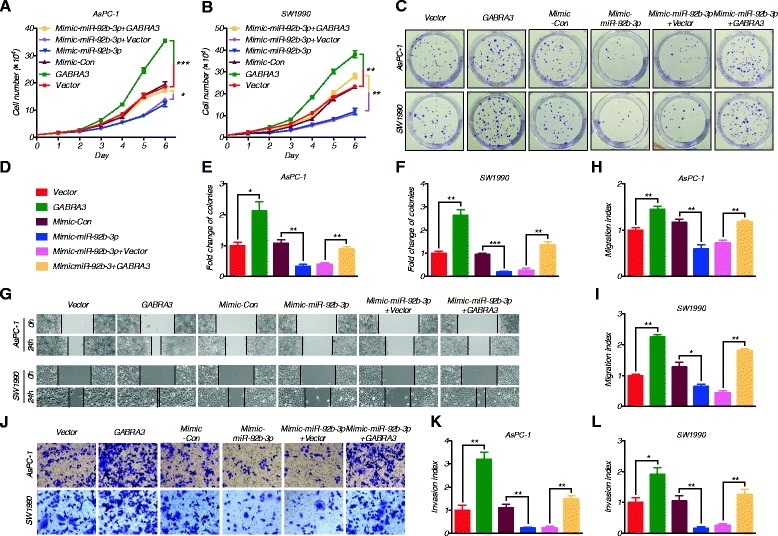
Gabra3 restoration reversed the miR-92b-3p-mediated inhibition of cell growth, migration and invasion in PC. **a**-**f** MTS and colony formation assays were performed to assess proliferation in PC cells transfected with miR-92b-3p alone or co-transfected with miR-92b-3p and *GABRA3*. **g-i** Wound healing assays were conducted to evaluate the motility of PC cells transfected with miR-92b-3p alone or co-transfected with miR-92b-3p and *GABRA3*. **j-l** Transwell assays were conducted in PC cells transfected with miR-92b-3p alone or co-transfected with miR-92b-3p and *GABRA3*. All *n* = 3; bar, SEM; *P < 0.05; **P < 0.01; ***P < 0.001; Student’s t test

Consistent with our previous findings in breast cancer cells, reduced Gabra3 expression levels also led to decreased cell numbers and colony formation ability in AsPC-1 and SW1990 cells (Fig. 5a-e). Similarly, PC cells with lower Gabra3 expression levels had suppressed cell migration and invasion capacities (Fig. 5f-k). At the same time, we found that altered Gabra3 expression levels had no effects on cell apoptosis (Fig. 5l-m). To better understand the reliance of miR-92b-3p on Gabra3 in modulating the biological behavior of PC cells, we then overexpressed *GABRA3* and miR-92b-3p and examined cell proliferation, migration and invasion abilities. Interestingly, *GABRA3* and miR-92b-3p co-overexpression attenuated the tumor inhibitory role of miR-92b-3p, as shown by increased proliferation (Fig. 6a-f), migration (Fig. 6g-i) and invasion (Fig. 6j-l) in both AsPC-1 and SW1990 cells. Taken together, these data imply that miR-92b-3p likely suppressed PC cell proliferation and metastasis through regulating Gabra3.

### The miR-92b-3p/Gabra3 axis regulated the AKT/mTOR and JNK pathways to modify PC proliferation and metastasis

**Fig. 7:**
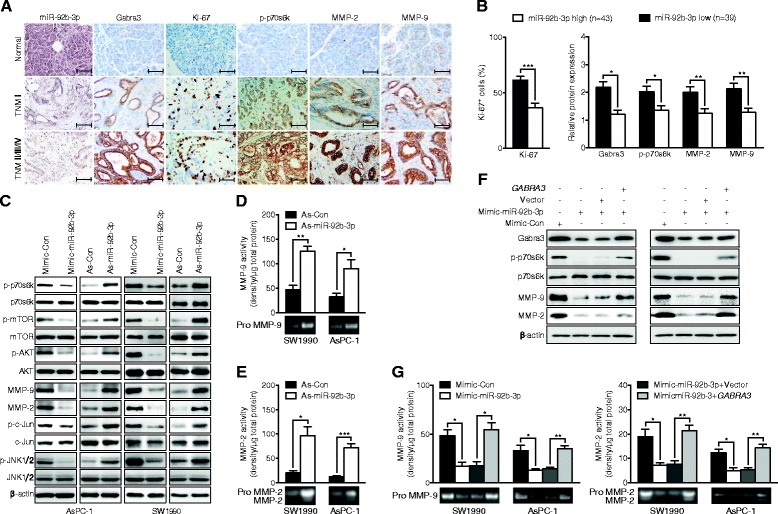
miR-92b-3p/Gabra3 interaction regulates PC cell proliferation and invasion via the AKT/m-TOR and JNK pathways. **a** Clinical specimens of normal pancreatic epithelium and PC with low and high TNM stages were stained for miR-92b-3p, Gabra3, Ki-67, p-p70s6k, MMP-2 and MMP-9. Representative images from a tissue microarray are shown. Original magnification, ×400; Scale bars: 50 μm. **b** Association analyses of miR-92b-3p levels and the number of Ki-67^+^ cells and the levels of p-p70s6k, MMP-2 and MMP-9 in 82 FFPE PC tissues. **c** Immunoblotting analyses of the levels of proteins in the AKT/m-TOR and JNK pathways in PC cells after transfection with miR-92b-3p mimic, inhibitor, or negative control. **d-e** MMP-2 and MMP-9 activities were measured by gelatin zymography in AsPC-1 and SW1990 cells after transfection with miR-92b-3p mimic, inhibitor, or negative control. **f** Immunoblotting analyses to detect p-p70s6k, MMP-2 and MMP-9 protein expression in PC cell lines co-transfected with the miR-92b-3p mimic and *GABRA3* construct. **g** Gelatin zymography analyses to detect MMP-2 and MMP-9 activities in PC cell lines co-transfected with miR-92b-3p mimic and *GABRA3* construct. All *n* = 3; bar, SEM; *P < 0.05; **P < 0.01; ***P < 0.001; Student’s t test

The AKT/mTOR and JNK pathways play vital roles in regulating proliferation and metastasis in many cancers. Indeed, Gabra3, AKT/mTOR and JNK pathway markers (p-p70s6k, MMP-2 and MMP-9) were obviously increased in PC patients with advanced TNM stages according to IHC analyses (Fig. 7a). We also noted that tumors with high miR-92b-3p expression levels often had a reduced number of Ki-67^+^ (cell proliferation marker) positive cells (Fig. 7a-b). Inverse relationships between miR-92b-3p and Gabra3, as well as p-p70s6k, MMP-2 and MMP-9, were also identified (Fig. 7a-b). Consistently, AsPC-1 and SW1990 cells overexpressing miR-92b-3p had decreased p-AKT, p-mTOR and p-p70s6k levels, as well as decreased p-JNK1/2, p-c-Jun, MMP-2 and MMP-9 protein levels (Fig. 7c). Consistently, gelatin zymography assays showed that MMP-2 and MMP-9 enzymatic activities were increased in PC cells expressing low levels of miR-92b-3p; however, their activities were decreased in PC cells overexpressing miR-92b-3p (Fig. 7d-e). In addition, Gabra3 overexpression abolished these regulatory effects of miR-92b-3p in both AsPC-1 and SW1990 cells (Fig. 7f-g), further supporting that miR-92b-3p regulates Gabra3.

### MiR-92b-3p inhibited cancer cell proliferation and metastasis in vivo

**Fig. 8:**
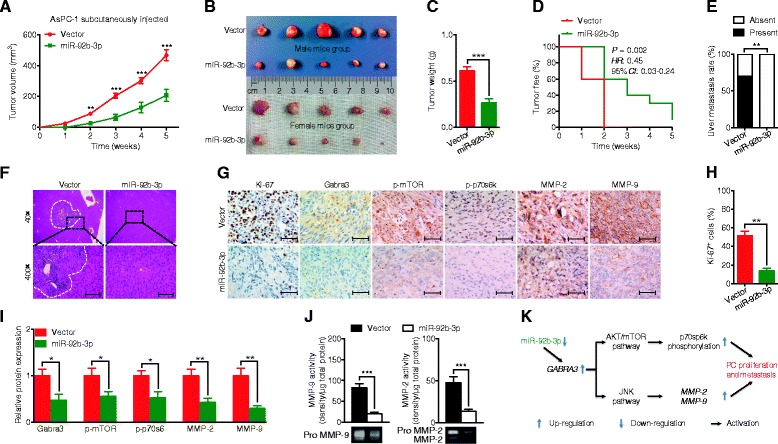
miR-92b-3p inhibited cell growth and metastasis in PC in vivo. **a**-**c** Tumor growth curve and tumor weights after AsPC-1 cells stably expressing miR-92b-3p or empty vector were injected into nude mice. (Each group, *n* = 10) (**d**) Comparison of the tumor formation frequency for PC cells stably expressing miR-92b-3p or empty vector. The *P* value was assessed by log-rank test. **e** Effects of miR-92b-3p on tumor metastases in nude mice. **f** H&E staining of the liver after injection of AsPC-1 cells transfected with miR-92b-3p or empty vector. Scale bars: 50 μm. **g-i** Immunohistochemistry and qPCR analyses to detect the number of Ki-67^+^ cells and the protein and mRNA levels of Gabra3, MMP-2 and MMP-9 in tumor xenografts from the miR-92b-3p overexpression and control groups. Representative images from 10 separate samples are shown. Original magnification, ×400; Scale bars: 50 μm. **j** Gelatin zymography analyses to detect MMP-2 and MMP-9 activities in tumor xenografts from the miR-92b-3p overexpression and control groups. **k** Proposed model for how miR-92b-3p regulates PC cell proliferation and metastasis by targeting Gabra3 via the AKT/mTOR and JNK pathways. We propose that miR-92b-3p interacts with Gabra3 via its mRNA 3′-UTR. Then, Gabra3 stimulates the AKT/mTOR and JNK pathways; these effects increase the phosphorylation of p70sp6k as well as the protein levels and activities of MMP-2 and MMP-9, thus increasing proliferation and metastasis in PC. Bar, SEM; *P < 0.05; **P < 0.01; ***P < 0.001; Student’s t test or Fisher’s exact test

PC xenograft mouse models were then used to determine the function of miR-92b-3p in vivo. Consistent with the in vitro findings, AsPC-1 cells overexpressing miR-92b-3p had slower tumor growth rates and lower tumor volumes, tumor weights, and tumor formation frequencies than the control group (Fig. 8a-d). To study the role of miR-92b-3p in modulating PC metastasis in vivo, all the livers of the xenograft nude mice were stained with hematoxylin-eosin (HE) to assess metastasis lesions. While 70% mice in the control group had liver metastases, they were rarely detected in the group overexpression miR-92b-3p (Fig. 8e-f). In addition, the tumors that formed due to miR-92b-3p overexpression also had fewer Ki-67^+^ cells and lower Gabra3, p-mTOR, p-p70s6k, MMP-2 and MMP-9 expression levels than those in the control group (Fig. 8g-j). Taken together, our data suggest an important role for miR-92b-3p in suppressing PC growth and metastasis in vivo.

## Discussion

In recent years, miRNA profiling and functional assays of various types of tumors have provided increasing evidence supporting the critical role of miRNAs in tumorigenesis and tumor progression [22, 23]. In our study, we found that miR-92b-3p levels were obviously reduced in PC tissues and cell lines and correlated well with the prognosis of PC patients. Moreover, miR-92b-3p overexpression inhibited proliferation, migration and invasion in PC cells, but miR-92b-3p knockdown yielded opposite effects in vitro and in vivo. In an effort to identify the downstream players, we found that miR-92b-3p directly regulated Gabra3 expression to modify the biological behavior of PC cells. The oncogenic regulatory role of the miR-92b-3p/Gabra3 axis was further demonstrated via modulation of the AKT/mTOR and JNK regulatory pathways. These results improve our understanding of PC-related miRNAs and support the development of miR-92b-based therapies for the treatment of PC.

By directly targeting the 3′-UTR of genes, miRNAs can suppress the expression of genes that are necessary for cell proliferation and metastasis signaling pathways [5]. The dysregulation of miR-92b-3p has been reported in several types of tumors, but these results are contradictory. The oncogenic roles of miR-92b-3p have been demonstrated primarily in regulating glioblastoma development [24, 25]. For example, it was suggested that miR-92b-3p overexpression increased viability in glioblastoma cells through repressing the TGF-beta/smad3/p21 signaling pathway [24]. In another report, miR-92b-3p targeted the PTEN/Akt signaling pathway to suppress proliferation, invasion and migration and stimulate apoptosis in glioma cells [25]. However, anti-tumor effects of miR-92b-3p have also been reported [26, 27]. Higher miR-92b levels have been inversely correlated with lymph node metastasis and better esophageal squamous cell carcinoma prognosis [26]. Investigations regarding the molecular mechanisms have proposed that miR-92b-3p targets the integrin α6/Akt axis or RAB23, a member of the Ras-related small GTPase family [27]. In our study, we used both in vitro and in vivo assays to confirm the tumor inhibitory role of miR-92b-3p in PC development. Because one miRNA can control multiple genes by binding to the 3′-UTR of target mRNAs, the combined biological effects are largely influenced by the specific genetic background of the types of cells or tumors in which the miRNA is expressed. Thus, on the one hand, the seemly distinct functions of miR-92b-3p in different types cancers reflect the intrinsic complexities and diversities of tumor biology; on the other hand, these distinct functions remind us that the biological roles of miRNAs could be context-dependent and that caution should be exercised when using miRNA therapies in clinical patients.

Previous studies have found that miR-92b-3p can control cell cycle progression through targeting *CDKN1C* [28]. However, among the nine candidate genes (including *CDKN1C*) predicted by TargetScan Release 7.0*,* only *GABRA3* had changes similar to the altered miR-92b-3p expression levels. GABA and GABA receptors are highly expressed in brain tissues and frequently associated with nervous system abnormalities [29]. Moreover, the aberrant expression of GABA receptors has also been found in multiple malignant tumors, which implies the importance of the GABAergic system in their pathogenesis and malignant transformation [30]. Recent evidence suggests that dysregulated Gabra3, one of of the subunits of the GABA receptor, can regulate cancer development, such as lung cancer [31]. Our previous study also confirmed that Gabra3 can promote breast cancer cell migration, invasion and metastasis by activating the AKT signaling pathway [20]. Hyperactivated oncogenic pathways, including the AKT/mTOR and JNK pathways, are important drivers of the malignant transformation of cancer cells [32]. In the present study, we found that increased Gabra3 levels were tightly correlated with increased tumor sizes, accelerated lymph node metastasis and advanced TNM stages and poor prognosis in PC patients. MiR-92b-3p overexpression decreased the levels of proteins related to the AKT/mTOR and JNK pathways, and this function of miR-92b-3p could occur through direct or indirect mechanisms. However, Gabra3 overexpression abolished the regulatory effects of miR-92b-3p on the AKT/mTOR and JNK pathways. Combined with two other breast cancer and lung adenocarcinoma studies indicating the influence of Gabra3 on the AKT/mTOR, and JNK pathways [20, 31], we suggest that the regulatory role of miR-92b-3p in the AKT/mTOR and JNK pathways might be indirect and Gabra3-dependent.

Matrix metalloproteinases (MMPs), a family of zinc-dependent enzymes that digest and degrade components of the extracellular matrix, are involved in cancer metastasis, including detachment, invasion, intravasation and extravasation, as well as angiogenesis and lymphangiogenesis [33]. MMP-2 and MMP-9 are important downstream players that influence the oncogenic role of the JNK pathway. A previous study shows that Gabra3 induced MMP-2 and MMP-9 expression through activating the JNK signaling pathway, which enhanced lymphatic metastasis in lung adenocarcinoma [31]. In our study, increased enzymatic activities and levels of MMP-2 and MMP-9 were found when miR-92b-3p expression was decreased; these effects could accelerate the migration and metastasis rates in PC via Gabra3. Compared with the variable targets that might be regulated by miRNAs, the oncogenic pathways required for cancer cell proliferation, invasion and metastasis have more in common. Our results suggest that reduced miR-92b-3p levels increased Gabra3 levels, which led to AKT/mTOR and JNK pathway activation in PC cells. These results seem to contradict previous findings showing that increased miR-92b-3p levels targeted the PTEN/Akt signaling pathway in gliomas [25]; however, these results probably also reflect the complicated and coordinated network that exists in cancer development, which could ultimately promote the malignant progression of cancers.

MiRNA-related studies have shown the increasing importance of the regulatory role of miRNAs. Current investigations suggest that miRNAs can be regulated at four levels [34]: epigenetic regulation, including methylation and acetylation; transcriptional regulation via factors such as c-Myc and P53; post-transcriptional regulation of miRNA biogenesis by Drosha and Dicer; and degradation via exoribonuclease. It has been suggested that phosphorylated ΔNp63α can up-regulate miR-92b-3p in squamous cell carcinomas [35]. Another study of epithelial ovarian cancer revealed that miR-92b could be regulated by aldehyde dehydrogenase 1 family member A2 (ALDH1A2) and protocadherin 9 (PCDH9) [36]. As with pancreatic cancer, the genes involved in regulating miR-92b remain unknown. Future investigations are needed to answer this question.

## Conclusions

Our results show that miR-92b-3p expression is down-regulated in PC tissues and cell lines and inversely related to clinical tumor size, lymph node metastasis, TNM stage and poor prognosis. In addition, the in vivo and in vitro experiments reveal that increased expression levels of miR-92b-3p suppress cell proliferation, migration, and invasion by targeting *GABRA3* in PC cells. This study revealed a novel tumor-suppressing mechanism in PC, indicating that miR-92b-3p may serve as a diagnostic and prognostic biomarker in PC patients. Further studies of PC are needed to define the function of the miR-92b-mediated molecular signaling pathway in controlling PC initiation and progression.

## Methods

### Tissue samples

PC and CNP (> 5 cm away from the PC tissues) tissues were retrospectively obtained from 46 patients who underwent surgical resection at the Department of Biliary-Pancreatic Surgery (Renji Hospital) from January 2012 to December 2014. All patients were confirmed by two pathologists as having pancreatic ductal adenocarcinoma. Tissue microarrays of 82 primary PC samples were obtained from Shanghai OutdoBiotech Ltd. All of the clinico-pathological features of the 82 PC patients were collected and are shown in Additional file 1: Table S1. The sample collection from and feature analyses of PC patients were approved by the Ethical Committee of Renji Hospital, Shanghai Jiao Tong University School of Medicine, and informed written consent was obtained from each patient.

### Cell culture and transfection

A normal pancreatic cell line (HPDE) and four cancer cell lines (CAPAN-2, AsPC-1, SW1990 and PANC-1) were used in this study. All the cell lines were purchased from Cell Resource Center of Shanghai Institutes for Biological Sciences, Chinese Academy of Sciences (Shanghai, China), and were cultured in DMEM containing 10% fetal bovine serum (Gibco, Grand Island, NY, USA) in a humidified atmosphere of 5% CO_2_ at 37 °C. For the inhibition and overexpression of miR-92b-3p and Gabra3, AsPC-1 and SW1990 cells were cultured to 60–70% confluence and then transfected with a miR-92b-3p mimic, miR-92b-3p inhibitor, *GABRA3* overexpression vector, *GABRA3* shRNAs (GCTGAAGTGGTTTATTCTTGG and GCTCTTTGCCATATTCAATCT) or respective controls (GenePharma, Shanghai, China) using Lipofectamine 2000 (Invitrogen, Carlsbad, CA, USA) according to the manufacturer’s instructions. After 24 or 48 h, the cells were collected for subsequent experiments. The human miR-92b-3p construct was created by inserting the coding sequence (CDS) of miR-92b-3p into the pCDH-CMV-MCS-EF1-copGFP vector (System Biosciences, Palo Alto, CA, USA). AsPC-1 cells stably overexpressing miR-92b-3p were created according to a previously described procedure [37].

### Cell proliferation and apoptosis assays

Cell proliferation was determined by MTS assays (Promega, Madison, WI, USA). Briefly, cells were seeded in 96-well plates and cultured at 37 °C for 24 h before transfection. Next, the cells were transfected with the corresponding oligonucleotides. At different time points, the MTS solution (20 μl) was added to each well of the plate. Then, the plates were incubated at 37 °C for 1 h, and the absorbance at 450 nm was measured. After transfection with the corresponding oligonucleotides for 48 h, PC cell apoptosis was detected using an Annexin V-FITC/PI Apoptosis Detection Kit (BD Biosciences, Heidelberg, Germany) according to the manufacturer’s instructions. The cells were stained with FITC-conjugated Annexin V and propidium iodide (5 mg/ml) on ice for 30 min and then measured by flow cytometry (BD Biosciences). The data were analyzed using FlowJo 9.1 software (Treestar, Ashland, OR, USA).

### Colony formation assay

For the colony formation assays, transfected cells were plated and grown in six-well plates in triplicate at a density of 500 cells per well. The cells were allowed to grow for 14 days at 37 °C until colonies were visible. To visualize the colonies, they were fixed in 4% paraformaldehyde for 15 min and stained with a 0.1% crystal violet solution. To assess the cloning ability of the PC cells, the numbers of colonies were manually counted under a dissection microscope.

### In vitro migration and invasion assays

For the wound healing assays, PC cells were plated in 6-well plates and cultured at 37 °C for 24 h. Wounds were made in the monolayer of the cells using a standard 200-μl pipette tip. Cells were washed to remove debris and incubated in DMEM without FBS at 37 °C. Images were taken at 24 h after wounding. The wound area was measured, and the percentage of wound healing was estimated by using ImageJ software (NIH, Bethesda, MD, USA). Cell invasion assays were conducted using 24-well transwell chambers with 8.0-μm pore size polycarbonate membranes (Corning Inc., Corning, NY, USA). Then, cells (5 × 10^4^) were seeded on the top side of the membrane pre-coated with Matrigel (Corning Inc.). After incubation, the cancer cells inside the upper chamber were removed with cotton swabs. The invaded cells on the lower membrane surface were then fixed and stained with a 5% crystal violet solution. Three images of ten random fields of each membrane were captured, and the number of migratory cells was counted. The means of triplicate assays for each experimental condition were used. The migration and invasion index is defined as the ratio of the experimental group to the control group.

### Gelatin zymography

The activities of MMP-2 and MMP-9 were determined by Zymography Gel Kit (Invitrogen) according to the manufacturer’s instructions. Briefly, equal amounts of protein were separated using 10% SDS-PAGE co-polymerized with 0.1% gelatin as a substrate. After electrophoresis, the gels were renatured for 1 h at room temperature in zymogram renaturing buffer and incubated at 37 °C overnight in zymogram developing buffer. After Coomassie brilliant blue staining, the gels were destained until clear bands could be visualized. The activities of the MMPs were quantified as the band intensity/μg total protein.

### Dual luciferase reporter assay

The 3′-UTR fragment from *GABRA3* mRNA containing the predicted miR-92b-3p binding site was amplified by PCR and then cloned into a pGL3 dual-luciferase miRNA target expression vector (Promega) to form the reporter vector (*GABRA3*-WT). The putative binding site of miR-92b-3p in the Gabra3 3′-UTR was mutated by using a site-directed mutagenesis kit (TransGen Biotech, Beijing, China), and the new reporter vector was called *GABRA3*-MU. The miR-92b-3p mimic and control were co-transfected into AsPC-1 and SW1990 cells. A Renilla luciferase reporter plasmid was also co-transfected as an internal reference. After transfection for 48 h, the cells were lysed in passive lysis buffer, and Firefly and Renilla luciferase activities were tested using the Dual-Luciferase Reporter Assay System (Promega). The Firefly luciferase activity results were normalized to the Renilla activity results.

### qPCR analysis

Total RNA was extracted from the tissues and cells using TRIzol reagent (Invitrogen). After synthesizing cDNAs with a miRNA cDNA Synthesis Kit (Takara, Shiga, Japan) and a Reverse Transcriptase MMLV Kit (Invitrogen), the expression levels of miR-92b-3p and its target genes were analyzed using SYBR Premix Ex Taq (Takara) and an Applied Biosystems ViiA™ 7 Real-Time PCR System (Applied Biosystems, Foster City, CA, USA). Data were analyzed by using the 2^−ΔΔCT^ method and presented as relative to the *GAPDH* mRNA and *RNU6B* levels for miR-92b-3p. All primer sequences used for qPCR are listed in Additional file 1: Table S2.

### Western blot analysis

For protein isolation from cells, RIPA buffer with proteinase inhibitor cocktail was used. The protein concentration was measured by BCA assay. Standard Western blot techniques and a Bio-Rad ChemiDoc MP imaging system (Hercules, CA, USA) were used according to previously described procedures [37]. The primary antibodies used were as follows: Gabra3 (1:1000, ab72446, Abcam, Cambridge, UK), p-p70s6k (1:1000, 9204, CST, Danvers, MA, USA), p70s6k (1:1000, 2708, CST), p-mTOR-S2448 (1:1000, 5536, CST), mTOR (1:1000, 2972, CST), p-AKT-S473 (1:2000, 4060, CST), AKT (1:2000, 2920, CST), MMP-9 (1:1000, 13,667, CST), MMP-2 (1:1000, 87,809, CST), p-c-Jun (1:1000, 9164, CST), c-Jun (1:1000, 2315, CST), p-JNK1/2 (1:1000, 4668, CST), JNK1/2 (1:1000, ab4821, Abcam), and β-actin (1:2000, A5316, Sigma, St. Louis, MO, USA).

### ISH analysis

ISH was used to detect miR-92b-3p in tissue microarrays using digoxigenin-labeled sense and antisense miR-92b-3p probes. Slides were de-paraffined and rehydrated before incubation with Proteinase K at 37 °C for 15 min; then, the slides were washed three times with 0.1 M TBS/diethyl pyrocarbonate for 15 min. After incubation with 5× SSC solution at room temperature for 15 min, miR-92b-3p probes were added for hybridization at 50 °C overnight. Next, the sections were washed with graded-diluted SSC solutions at 50 °C for 30 min, followed by incubation with an antibody against digoxigenin (1:1000, Roche, Mannheim, Germany) at room temperature for 2 h. Finally, hybridization signals were visualized by NBT/BCIP (Sigma). The reaction was stopped by washing with water for 5 min. Slides were then counterstained with nuclear fast red, mounted using an aqueous solution, and photographed.

### IHC analysis

FFPE tissue microarrays were cut into 3-μm sections for immunohistochemistry. Slides were deparaffinized with xylene and hydrated with decreasing concentrations of an ethanol solution. Sections were treated with citrate buffer solution (Maixin Bio, Fuzhou, China) at 100 °C for 1 min for antigen retrieval and were permeabilized in 3% hydrogen peroxide for 10 min at 37 °C. Slides were then incubated with primary antibodies at 4 °C overnight, followed by incubation with secondary antibodies for 60 min at 37 °C. Finally, 3,3-diaminobenzidine tetrahydrochloride was used as coloring reagent, and hematoxylin was used as a counterstain for nuclei. The stained fields were photographed using a light microscope equipped with a camera (Olympus, Tokyo, Japan). The primary antibodies used for IHC were as follows: Gabra3 (1:200, G4291, Sigma), p-p70s6k (1:400, 9204, CST), MMP-9 (1:300, 13,667, CST), MMP-2 (1:200, ab97779, Abcam) and Ki-67 (1:1000, ab833, Abcam). Semi-quantitative scoring of ISH and IHC was based upon the staining intensity (I: negative, 0; weak, 1; moderate, 2; and intense, 3) and the percentage of positive-stained cells (P: 0–5%, score of 0; 6–35%, score of 1; 36–70%, score of 2; and >70%, score of 3) to obtain a final score (Q) defined as the product of I × P. Low levels of expression were defined as Q < 4, and high levels of expression were defined as Q ≥ 4. Two senior pathologists performed the scorings independently in a blinded manner.

### Xenograft tumor study

All animal studies were conducted strictly in accordance with the guidelines of the Animal Care and Use Committee of Shanghai Jiao Tong University. Four-week-old nude mice (15–20 g) were purchased from the Shanghai Laboratory Animal Research Center (Shanghai, China). To evaluate cell proliferation in vivo*,* miR-92b-3p-overexpressing and negative control AsPC-1 cells (2 × 10^6^) were subcutaneously injected into the right and left hind footpads of the mice (males, *n* = 5; females, *n* = 5). Tumor growth curves were measured weekly with a caliper from the 1st week to the 5th week. Tumor volume analyses were based on the equation: V = (length × width^2^) / 2. For the tumor metastasis study, miR-92b-3p-overexpressing and negative control AsPC-1 cells (1 × 10^6^) were transplanted onto the head or body of the pancreas of the mice (males, *n* = 5; females, *n* = 5). After five weeks, all mice were sacrificed, and the tumors were incised and analyzed for weight and IHC. All mouse livers were also harvested to count the number of metastases.

### Statistical analysis

Statistical analyses were conducted using SPSS17.0 software (IBM, Chicago, IL, USA). Data are expressed as the mean ± standard error of the mean (SEM). Group comparisons of normally distributed data were performed using unpaired Student’s t tests (two-tailed). Dichotomous variables were compared using χ^2^ tests. Pearson χ2 tests were used to analyze the association between miR-92b-3p expression levels and *GABRA3* expression levels. Survival probabilities were estimated based on the Kaplan-Meier method and analyzed by log-rank test. *P* < 0.05 was considered statistically significant.

## Additional file

Additional file 1: Table S1. Correlation between miR-92b-3p expression and clinico-pathologic features. **Table S2.** Primer for qPCR in this study. (DOCX 19 kb)

## Abbreviations

ALDH1A2: aldehyde dehydrogenase 1 family member A2
CDS: the coding sequence
CNP: corresponding noncancerous pancreatic
FFPE: formalin-fixed paraffin-embedded
GABA: gamma-aminobutyric acid
HE: hematoxylin-eosin
IHC: immunohistochemistry
ISH: in situ hybridization
miRNAs: microRNAs
MMPs: Matrix metalloproteinases
PC: pancreatic cancer
PCDH9: protocadherin 9
qPCR: quantitative PCR
SEM: standard error of mean
TNM: tumor/node/metastasis
UTR: untranslated region
