# Supplementary material for: miR-92b-3p acts as a tumor suppressor by targeting Gabra3 in pancreatic cancer
Source: Mol Cancer. 2017 Oct 27;16:167. doi: 10.1186/s12943-017-0723-7 (PMC5659029; doi:10.1186/s12943-017-0723-7)
Supplement: Additional file 1: Table S1. — Correlation between miR-92b-3p expression and clinico-pathologic features. Table S2. Primer for qPCR in this study. (DOCX 19 kb) [file 12943_2017_723_MOESM1_ESM.docx]

**Additional File 1**

**Table 1.** Correlation between miR-92b-3p expression and clinico-pathologic features

| Characteristics | No. of patients (n=82) | Low expression (n=39) | High expression (n=43) | χ^2^ | *P value* |
| --- | --- | --- | --- | --- | --- |
| Gender |  |  |  |  |  |
| Female | 32 (39.0) | 14 (35.9) | 18 (41.9) | 0.31 | 0.580 |
| Male | 50 (61.0) | 25 (64.1) | 25 (58.1) |  |  |
| Age, y |  |  |  |  |  |
| ≤ 60 | 38 (46.3) | 17 (43.6) | 21 (48.8) | 0.23 | 0.634 |
| > 60 | 44 (53.7) | 22 (56.4) | 22 (51.2) |  |  |
| Tumor size, cm |  |  |  |  |  |
| ≤ 5 cm | 52 (63.4) | 20 (53.1) | 32 (74.4) | 4.72 | **0.030** |
| > 5 cm | 30 (36.6) | 19 (48.7) | 11 (25.6) |  |  |
| Lymph node metastasis |  |  |  |  |  |
| Absent | 50 (61.0) | 17 (43.6) | 33 (76.7) | 9.45 | **0.002** |
| Present | 32 (39.0) | 22 (56.4) | 10 (23.3) |  |  |
| TNM stage |  |  |  |  |  |
| Ⅰ | 40 (48.8) | 13 (33.3) | 27 (62.8) | 7.10 | **0.008** |
| Ⅱ, Ⅲ, and Ⅳ | 42 (51.2) | 26 (66.7) | 16 (37.2) |  |  |

**Table 2.** Primer for qPCR in this study

| Genes (*Homo sapiens*) | Primers | Sequences |
| --- | --- | --- |
|  | Anchor RT primer | CGACTCGATCCAGTCTCAGGGTCCGAGGTATTCGATCGAGTCGCACTTTTTTTTTTTTV |
| miR-92b-3p | Forward | 5'-CCGCCCTGCTCACGTTAT-3' |
|  | Reverse | 5'-AGTCTCAGGGTCCGAGGTATTC-3' |
| *RNU6B* | Forward | 5'-CTCGCTTCGGCAGCACA-3' |
|  | Reverse | 5'-AACGCTTCACGAATTTGCGT-3' |
| *GABRA3* | Forward | 5'-TCTGGACGGCTATGACAACC-3' |
|  | Reverse | 5'-TTCATCATGCCATGTCTGCC-3' |
| *CDKN1C* | Forward | 5'-CACTCGGGGATTTCGGGAC-3' |
|  | Reverse | 5'-CTTGGAGAGGGACACGGC-3' |
| *LATS2* | Reverse | 5'-GCAGAAGTGAACCGGCAAAT-3' |
|  | Forward | 5'-AATCTGCTCATTCCTCGGGT-3' |
| *DUSP6* | Reverse | 5'-TGATAGATACGCTCAGACCCG-3' |
|  | Forward | 5'-TTGATGGCCGACTCGATGT-3' |
| *NIPBL* | Forward | 5'-CAGGGAGAAAAGCATGCAGA-3' |
|  | Reverse | 5'-GCTTGTCTGTGGTGGCAC-3' |
| *ITGA6* | Forward | 5'-ACTGTGAGCTCGGAAATCCT-3' |
|  | Reverse | 5'-GTTTAGCAACTCCCGAGACC-3' |
| *TWIST1* | Reverse | 5'-CTCGGTCTGGAGGATGGAG-3' |
|  | Forward | 5'-CCACGCCCTGTTTCTTTGAA-3' |
| *RAB23* | Reverse | 5'-CCGATCCCAAGTTCCAGCTA-3' |
|  | Forward | 5'-TTCCAACTGCTCCATTCCCT-3' |
| *GAPDH* | Forward | 5'-GAAGGTGAAGGTCGGAGTC-3' |
|  | Reverse | 5'-GAAGATGGTGATGGGATTTC-3' |
